# The effects of prolonged stress exposure on the brain of rats and insights to understand the impact of work-related stress on caregivers

**DOI:** 10.3389/fnbeh.2023.1288814

**Published:** 2023-11-30

**Authors:** Jean Marc Pujo, Dewi Yunia Fitriani, Hajer Ben Saad, Marwa Ghariani, Amel Dghim, Manel Mellouli, Antoine Burin, Remi Mutricy, Stephanie Houcke, Ariane Roujansky, Muchtaruddin Mansyur, Flaubert Nkontcho, Bertrand de Toffol, Ibtissem Ben Amara, Hatem Kallel

**Affiliations:** ^1^Emergency Department, Cayenne General Hospital, Cayenne, French Guiana; ^2^Community Medicine Department, Faculty of Medicine Universitas Indonesia, Jakarta, Indonesia; ^3^Occupational Medicine Specialist Program, Faculty of Medicine, Universitas Indonesia, Jakarta, Indonesia; ^4^Occupational and Environmental Health Research Center, IMERI, Faculty of Medicine, Universitas Indonesia, Jakarta, Indonesia; ^5^Laboratory of Medicinal and Environment Chemistry, Higher Institute of Biotechnology, University of Sfax, Sfax, Tunisia; ^6^Laboratory of Molecular and Cellular Screening Processes (LPCMC), LR15CBS07, Center of Biotechnology of Sfax, University of Sfax, Sfax, Tunisia; ^7^Laboratory of Anatomopathology, CHU Habib Bourguiba, University of Sfax, Sfax, Tunisia; ^8^Intensive Care Unit, Cayenne General Hospital, Cayenne, French Guiana; ^9^Pharmacy Department, Cayenne General Hospital, Cayenne, French Guiana; ^10^Neurology Department, Cayenne General Hospital, Cayenne, French Guiana; ^11^Tropical Biome and Immunopathology CNRS UMR-9017, Inserm U 1019, Université de Guyane, Cayenne, French Guiana

**Keywords:** stress, healthcare, oxidative stress, acetylcholine, apoptosis, corticosterone, brain damage

## Abstract

**Introduction:**

Stress exposure is a significant concern in the healthcare sector. This animal model study aims to reproduce caregivers’ working conditions and determine their impact on the brain.

**Method:**

Twenty-four healthy male rats of the Wistar strain were divided into four groups. Three groups were submitted each to one stressor for 21 days, while the fourth group was used as a control. Stressors were food and water deprivation (FW), permanent illumination (PI), and forced swimming (FS). At the end of the experiment, rats were euthanized, and stress biomarkers, biological parameters, and DNA damage were measured.

**Results:**

Prooxidant biomarker rates increased in the different groups (+50 to +75%) compared to the control (*p* < 0.0001). Urinary corticosterone rates increased in all stressed animals, mainly in the PI group, with changes of up to +50% compared to the control group. Acetylcholinesterase levels decreased to −50% (*p* < 0.0001 for the three exposed groups). Total ATPase, (Na^+^/K^+^)-ATPase, and Mg^2+^-ATPase activities decreased in all stressed groups. The percentage of brain cell congestion and apoptosis was 3% for the FW group (*p* < 0.0001), 2% for the PI group (p < 0.0001), and 4% for the FS group (p < 0.0001) compared to the control (0.8%). DNA damage was observed in all exposed groups. Finally, we noticed behavioral changes and a depression-like syndrome in all stressed rats.

**Conclusion:**

Stressful conditions such as the working environment of caregivers can trigger several pathophysiological processes leading to oxidative, neurochemical, and hypothalamic–pituitary–adrenal disorders. These changes can progress to cell damage and apoptosis in the brain and trigger psychological and physical disorders.

## Graphical abstract

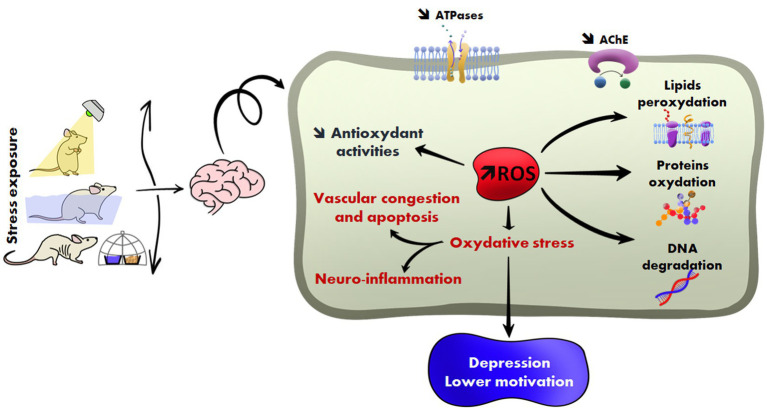

## 1 Introduction

Work-related stress is a significant concern in the healthcare sector due to its negative impact on workers’ health and performance (Koenen et al., 2017; Pujo et al., 2022). It represents a physical and emotional response when the job demands exceed the capabilities or resources of the person (Pujo et al., 2021, 2022). The weight of medical consequences calls for continuous stress monitoring at work to pinpoint its sources, prevent burnout, and adopt coping strategies to eliminate or reduce it until it reaches an acceptable level (Yang et al., 2018).

The holistic vision of health (or the absence of disease) includes physical and psychological dimensions (Sharpe and Naylor, 2016). The WHO emphasizes the role of the environment and includes an additional dimension in the causality of the disease, which is intended to be “harmonic” healthy conditions (Shadloo et al., 2016). Individuals can be in tune with their values, deal with daily pressures, engage in fruitful and productive work, and have the ability to contribute positively to their community (Shadloo et al., 2016). In this context, the relationship between work-related stress and mental health has interested many researchers as a factor affecting cognition. Cunningham and Regan (2016) pointed out that the impact of work-related stress on mental health is pervasive, regardless of age and industry.

Major depression and other mental disorders could result from disturbances in the metabolism of some neurotransmitters. Acetylcholine (ACh) is a neurochemical synaptic transmitter that plays a pivotal role in cognitive and brain functions. There is strong evidence that increased ACh leads to the exacerbation of depression symptoms or other affective disorders (Overstreet and Janowsky, 1992; Abdulla and Picciotto, 2023). Glucocorticoids are involved in the stress response. In mice, the main glucocorticoid is corticosterone, while in humans, it is cortisol (Nandam et al., 2019). Several studies have shown a correlation between the severity of symptoms and cortisol levels (Zobel et al., 2001).

The brain tissue is highly sensitive to oxidative stress because of its poor shield of antioxidant enzymes and low regeneration power compared to other tissues (Chirino and Pedraza-Chaverri, 2009). The enzymatic antioxidants play a fundamental role in scavenging reactive oxygen species (ROS) and preventing their formation (Veerappan et al., 2004). These enzymes convert active oxygen molecules into non-toxic compounds (Pradeep et al., 2008). Superoxide dismutase (SOD) is a ubiquitous enzyme that protects aerobic cells against oxidative stress. It is primarily a mitochondrial enzyme usually found in the plasma membrane. Catalase is a tetrameric heme protein that undergoes alternative divalent oxidation and reduction at its active site in the presence of hydrogen peroxide. As a substrate for glutathione peroxidase (GPx), reduced glutathione protects cellular constituents from the damaging effects of peroxides and other ROS. GPx catalyzes hydroperoxide reactions with reduced glutathione to form glutathione disulfide and reduced hydroperoxide products. Over-production of free radicals and disturbances in the capacity of antioxidant defense have been involved in a large number of diseases (Ben Saad et al., 2017).

Finally, studies have explored the relationship between redox turbulence and neuropsychiatric disorders. Indeed, the brain is rich in lipids, high in energy and oxygen consumption, and has a low antioxidant defense capacity, making it vulnerable to ROS-mediated peroxidation and reactive nitrogen species (RNS) (Salim, 2017). Together, they can cause severe lipid, protein, and DNA damage. Moreover, psychiatric disorders have been observed in the case of increased ROS levels (Anderson and Maes, 2014). High levels of some prooxidant or antioxidant enzymes and low levels of exogenous antioxidants are associated with depression. Lipid peroxidation is caused by an increased concentration of proinflammatory cytokines that produce free radicals (Vaváková et al., 2015).

This study was designed to investigate the effects of redox turbulence, acetylcholine imbalance, and corticosterone release using three kinds of stressors reproducing work conditions in health facilities. The experience gained from laboratory rats was expected to provide valuable information to highlight the impact of poor working conditions on caregivers’ health.

## 2 Materials and methods

### 2.1 Animals and establishment of work-related stress models

Twenty-four healthy male rats of the Wistar strain (age 8–10 weeks; weight 150 ± 10 g) were used in this study. All animals were housed in pre-bedded polyethylene cages with standard laboratory conditions (temperature 25 ± 2°C and 12 h light/dark cycle). Animals had free access to commercial pellet diets (SNA, Sfax, Tunisia) and water. The experimental procedures were carried out in compliance with the European Union legislation (Directive 2010/63/EU, 2010) on the protection of animals used for scientific purposes (Directive 2010/63/EU of the European Parliament and of the Council of 22 September 2010 on the protection of animals used for scientific purposes Text, with EEA relevance, 2010) and approved by the local ethical committee (Protocol n° 09.0010/22).

Rats were randomly divided into four groups (*n* = 6 each). One group served as the no-stress subgroup (control). Rats in the stressed subgroups were subjected to 21 days of three forms of stress.

The FW group was exposed to stress from food and water restrictions.

The PI group was exposed to bright continuous light (24/7).

The FS group was subjected to forced swimming stress sessions.

#### 2.1.1 Food and water deprivation protocol

Our experimental protocol was carried out while fully respecting the food and water restriction guidelines in rodent experiments where restricted levels do not exceed 30% of food and water provided *ad libitum* (Kant et al., 1988; Voigt et al., 1996; Bert et al., 2006; Carlini et al., 2008; Conrad, 2010).

#### 2.1.2 **Permanent** illumination protocol

Animals were submitted to light stress by being exposed to bright continuous light given by a 100-W bulb 2.75 m above the center of the room-approximately 100 lx at the central floor level, and temperatures were held between 24 and 27°C during the experiment, with free access to food and water. Animals were subjected to an accommodation period to the room conditions and light cycle for 3 days before the beginning of the experiment. During this period, they were not handled (Dauchy et al., 2013).

#### 2.1.3 **Forced swim** protocol

Rats were subjected to 20 days of forced-swim stress sessions (4 days/5). During this period, rats were separately put into the water-filled forced-swim pool for 15 min daily (Kawabe, 2017). The rats were individually made to swim inside a vertical borosilicate glass jar (25 cm × 12 cm × 25 cm) containing water at 23 ± 1°C. The water depth was adjusted according to the rat’s size so that its hind legs did not touch the bottom of the container. When placed in the jar for the first time, the rats were initially highly active, vigorously swimming in circles and trying to climb the wall or dive to the bottom. After 15 min in the water, the rats were removed, wiped with a dry cloth, and allowed to dry before being returned to their home cages. The jar was emptied and washed thoroughly after testing for each rat.

### 2.2 Behavioral testing

**Figure 1:**
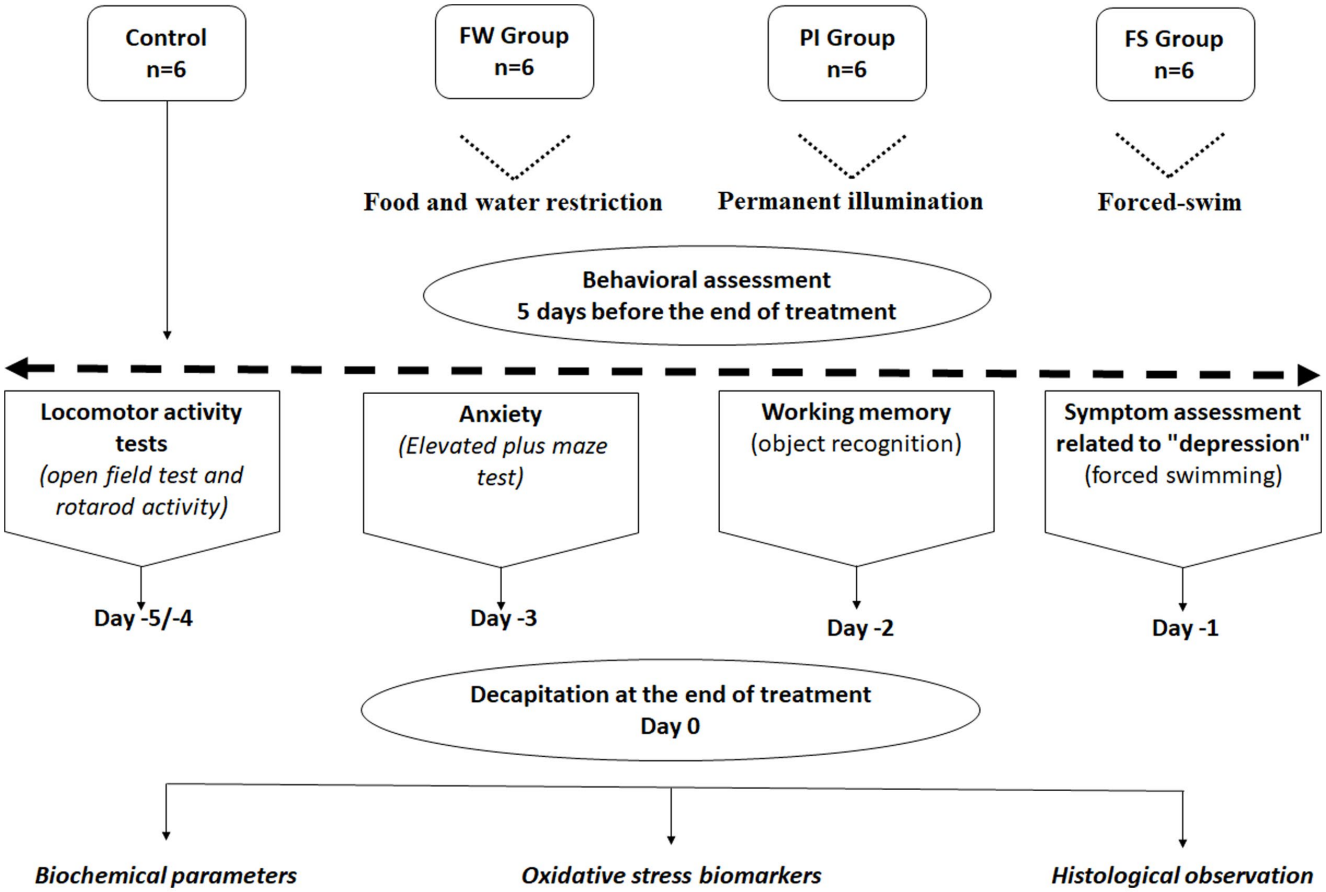
Establishment of work-related stress models.

A behavioral assessment was performed 5 days before the end of treatment. At day 21 of exposure, all rats were euthanized by decapitation (Figure 1). Four series of behavioral tests were implemented in the following order (Kawabe, 2017):

(1) locomotor activity tests (rotarod activity and open field test)
(2) anxiety (elevated plus maze test)
(3) working memory (object recognition)
(4) symptom assessment linked to “depression” (forced swimming)

These tests were established to explore the effects of the selected stressors on different groups of animals, including the control rats.

#### 2.2.1 *Locomotor* activity tests

##### 2.2.1.1 Rotarod activity

The ability of a rodent to maintain balance and keep pace with a rotating rod has been used to assess motor function and coordination activity in animals. Several versions of this test (commonly referred to as the rotarod test) have been described. Most of them require the rat to walk on a rotating rod of fixed diameter that increases in speed over a predetermined period until the animal can no longer maintain its position. The speed at which the rod rotates can be accelerated from 0 rpm to over 100 rpm over 3 min.

The principle of this test is that rats are first trained to walk on a rod rotating at a certain speed. Once the animals have learned this, their motor performance is evaluated. Animals experiencing impaired motor coordination are unable to cope with the rotating rod and will drop off when the rotation speed exceeds their motor coordination capacity. When the animal drops from the rod safely into its own lane, the time it takes to fall is automatically recorded.

Motor coordination and grip strength were assessed using a rotarod apparatus (Föhr Medical Instruments GmbH of Germany). Animals were exposed to the rotarod for 300 s before the training session to acclimate them to the apparatus. They were then placed on the rotating rod (diameter of 3 cm, speed 20 rpm) for 3 min. The time of rat falling from the rod was recorded (Fan et al., 2008).

##### 2.2.1.2 Open field test

The open field device was a square-shaped wood box, 80 cm in length and 60 cm in height. The floor was divided into 16 squares. It was placed in a quiet room with controlled temperature and ventilation. We recorded the number of squares in which each rat crossed with its paws (crossing); stood on its legs (rearing); and wiped, licked, or combed any part of its body (grooming). Each rat was placed in the center of the device, and the number of head dips and head dipping duration (in seconds) were recorded during a 5-min observation (Fan et al., 2008). A head dip was included if both eyes were steered down. Between two tests, the floor was cleaned with ethyl alcohol and permitted to dry (Sumathi et al., 2016).

#### 2.2.2 Anxiety assessment: the elevated plus maze test

The elevated plus maze test is a widely used behavioral assay to assess the anxiety symptoms and to define brain regions and mechanisms underlying anxiety-related behavior in rodents (Lamprea et al., 2001). It consists of two open arms crossed at right angles with two opposed arms of the same size. Two of the opposed arms were enclosed by walls 60 cm high, except for the central part where the arms crossed. The whole device is elevated 50 cm above the floor. We recorded the number of squares entered by an animal (which made it possible to estimate the distance run) as well as the exact place of occurrence of specific behaviors. The measures consisted of determining the number of locomotor activities, the time spent in open arms, and the time spent in closed arms (Montgomery and Monkman, 1955).

#### 2.2.3 Working memory: object recognition

Working memory is a property of the brain that supports successful attainment of behavioral goals that are being carried out by any of several systems, including sensory systems, those that underlie semantic and episodic memory, and motor systems. Object recognition is a neural mechanism that likely underlies working memory function (Hannesson et al., 2008).

The rats were first exposed to two equivalent objects, made of glass with a height of 12 cm and a maximum diameter of 4 cm, placed in a balanced fashion in diametrically positioned corner pairs of the open field. The rats were exposed to the objects for 5 min on 2 consecutive days. Thereafter, on the third day, one object was replaced by a novel glass object with equal height but a different shape and texture. The animals were allowed to explore the ‘old’ and ‘novel’ objects for 5 min. On the following day, the “old” object was displaced to the center of the open field, while the “novel” object remained at its original location. After each trial, the device and the objects were cleaned with water containing 0.1% acetic acid. The number of object contacts with forepaws or vibrissae, the total distance moved (cm), and the number of rearings on the walls and the inner parts of the field were scored (Hannesson et al., 2008).

#### 2.2.4 Depression symptom assessment: forced swimming (Porsolt test)

The forced swimming test is predictive of depressive-like behavior in rodents. It consists of two parts: an initial training period of 15 min and an actual test for 5 min 24 h later. The rats were individually forced to swim inside a vertical borosilicate glass jar (25 cm × 12 cm × 25 cm) containing water at 23 ± 1°C. The water depth was adjusted according to the rat’s size so that its hind legs did not touch the bottom of the container. The detailed steps to perform the forced swimming test were carried out according to Yankelevitch-Yahav et al. (2015).

### 2.3 Fatty acid analysis

Fatty acids were extracted from the cerebrum tissue, and fatty acid methyl esters (FAMEs) were prepared according to the method by Milinsk et al. (2008). After lipid extraction using the Soxhlet method and saponification, fatty acids were esterified. Samples were dissolved in 0.5 mL of hexane. Then, 0.2 mL of potassium hydroxide (2 N) in methanol was added for the fatty acid methylation process. The mixture was vortexed and centrifuged, and the upper phase containing fatty acid methyl esters was subjected to cute letter gas chromatography (GC) analysis. FAMEs were analyzed on a Shimadzu device (GC-17A) equipped with a polar capillary column (DB-WAX, 3.0 m length, 0.25 mm, 0.25 μm film thickness; Supelco). The oven temperature was programmed from an initial temperature of 150°C (0.5 min hold), then rising to 200°C at 6°C/min, to 230°C at 4°C/min, and held isothermal at 250°C for 15 min. Nitrogen was used as a carrier gas at a flow rate of 1 mL/min. The injection port and the flame ionization detector were maintained at 250°C. Identification was made by comparing retention times to those of authentic standards.

### 2.4 Brain preparation

At the end of the experiment, the cerebrum was quickly removed. Some samples were homogenized in Tris–HCl buffer pH 7.4 with an Ultra Turrax homogenizer and centrifuged at 10,000 × g for 15 min at 4°C. The resulting supernatants were used for various biochemical assays. Other samples were immediately fixed in a 10% formalin solution for histological studies.

### 2.5 Biochemical assays

#### 2.5.1 Protein quantification

Cerebrum protein contents were measured according to the method described by Lowry et al. (1951) using bovine serum albumin as a standard.

#### 2.5.2 Lipid peroxidation measurement

The brain malondialdehyde (MDA) concentration index of lipid peroxidation was determined spectrophotometrically according to the method of Draper and Hadley (1990). Briefly, 0.5 mL of cerebrum extract was mixed with 1 mL of trichloroacetic acid solution and centrifuged at 2500 g for 10 min. The resulting supernatant (0.5 mL) and 1 mL of a solution containing 0.67% thiobarbituric acid (TBA) were incubated for 15 min at 90°C and then cooled. The mixture was measured for absorbance at 532 nm using a spectrophotometer (Jenway UV6305, Essex, England). The MDA values were calculated using 1,1,3,3-tetraethoxypropane as standards and expressed as nanomoles of malondialdehyde/mg protein.

#### 2.5.3 Determination of protein carbonyl (PCO) content

PCO content in the cerebrum tissue was measured according to the method described by Reznick and Packer (1994). In brief, 100 mL of cerebrum extract was placed in glass tubes. Then, 500 mL of 10 mM 2,4 dinitrophenyl hydrazine (DNPH) in 2 N HCl was added. The tubes were incubated for 1 h at room temperature. Samples were vortexed every 15 min. Then, 500 mL of TCA (20%) was added, and the tubes were left on ice for 5 min followed by centrifugation for 10 min. The pellet of protein was washed twice with ethanol ethyl acetate (v/v). The final precipitate was dissolved in 600 mL of a 6 M guanidine hydrochloride solution and incubated for 15 min at 37°C. PCO was calculated based on the molar extinction coefficient of DNPH and was expressed as nanomoles per milligram of protein.

#### 2.5.4 Hydrogen peroxide (H_2_O_2_) assay

A hydrogen peroxide (H_2_O_2_) assay was performed using the ferrous ion oxidation xylenol orange (FOX-1) method. The FOX1 reagent consisted of 25 mM sulfuric acid, 250 mM ferrous ammonium sulfate, 100 mM xylenol orange, and 0.1 M sorbitol. In brief, 100 mL of extract was added to 900 mL of FOX1 reagent, vortexed, and incubated for 30 min at room temperature. Solutions were then centrifuged at 12.000 *g* for 10 min. The amount of H_2_O_2_ in the cerebrum extract was measured at 560 nm in a spectrophotometer and expressed as μmol/mg protein.

#### 2.5.5 Lipid hydroperoxides (LOOHs) assay

Lipid hydroperoxides (LOOHs) were quantified using a FOX assay as described by Jiang et al. (1992). The FOX2 reagent consisted of 90% methanol (v/v), 250 mM H_2_SO_4_ (v/v), 4 mM BHT, 250 mM ferrous ammonium sulfate hexahydrate, and 100 mM xylenol orange. The methanol, H_2_SO_4_, and BHT were mixed and stored at 4°C, whereas the iron and xylenol orange were added just prior to the addition of reagents to the samples. The mixture was vortexed and incubated for 30 min at room temperature. Then, 900 mL of FOX2 reagent was added to each sample, with the absorbance at 560 nm being recorded exactly 10 min after reagent addition. The amount of LOOH produced was calculated using a molar extinction coefficient of 4.59 × 104 mol.L^−1^.cm^−1^. Values are expressed as nmol/mg protein.

#### 2.5.6 Advanced oxidation protein products (AOPPs) determination

Advanced oxidation protein products (AOPPs) were assayed using the method described by Witko et al. (1992). Briefly, 0.1 mL of 1.16 M potassium iodide (KI) was added to the cerebrum homogenate, followed by 0.2 mL of acetic acid. The level of AOPP in the cerebrum tissue was calculated using an extinction coefficient of 261 mmol.L^−1^.cm^−1^ and expressed as μmol/mg protein.

#### 2.5.7 Superoxide dismutase (SOD) activity

Superoxide dismutase (SOD) activity was estimated according to Beauchamp and Fridovich (1971). The reaction mixture consisted of 50 mL of the cerebrum homogenate in Tris–HCl buffer (pH 7.4), 13 mM L-methionine, 75 mM Nitro Blue Tetrazolium (NBT), 0.1 mM EDTA, and 2 mM riboflavin. The developed blue color of the reaction was measured at 560 nm. The activity was expressed as units/mg protein.

#### 2.5.8 Glutathione peroxidase (GPx) activity

The activity of glutathione peroxidase (GPx) was measured according to Flohé and Günzler (1984). Briefly, 200 mL of the homogenized cerebrum was added to 200 mL of the reduced glutathione reductase (4 mM) and 100 mL of 100 mM phosphate buffer, with a pH value of 7.4. In the presence of nicotinamide adenine dinucleotide phosphate reduced form (NADPH), the oxidized reduced glutathione is immediately converted to the reduced form with a concomitant oxidation of NADPH/NADP^+^. The decrease in absorbance at 340 nm was determined. The enzyme activity was expressed as nmol of GSH oxidized/min/mg protein.

#### 2.5.9 Catalase (Cat) activity

Catalase (CAT) activity was assayed by the decomposition of hydrogen peroxide according to the method of Aebi (1984). The enzymatic reaction was initiated by adding an aliquot of 20 mL of the homogenized cerebrum and the substrate (H_2_O_2_) to a concentration of 0.5 M in a medium containing 100 mM phosphate buffer (pH 7.4). The enzyme activity was expressed as mmol H_2_O_2_ consumed/min/mg protein.

#### 2.5.10 Total glutathione (GSH) content

Total glutathione (GSH) in the cerebrum was determined by the method of Ellman (1959) based on the development of a yellow color when 5,5-dithiobis-2-nitrobenzoic acid (DTNB) was added to compounds containing sulfhydryl groups. Briefly, 500 μL of cerebrum homogenate in Tris–HCl buffer was added to 3 mL of 4% sulfosalicylic acid. The mixture was centrifuged at 3500 *g* for 10 min. Ellman’s reagent was added to 500 μL of supernatants. The absorbance was measured at 412 nm after 10 min. Total GSH content was expressed as nanomoles per milligram of protein.

#### 2.5.11 Acetylcholinesterase (AChE) activity

Acetylcholinesterase (AChE) activity in the cerebrum tissue was measured immediately in homogenates (Lombardi et al., 1999), using acetylthiocholine iodide as a substrate. The reaction mixture included phosphate buffer (0.1 M, pH 8), 0.075 M acetylthiocholine iodide, and 0.01 M DTNB. The hydrolysis of acetylthiocholine was measured at 412 nm through the release of the thiol compound, which reacted with DTNB. AChE activity was expressed as micromoles of substrate hydrolyzed per minute per milligram of protein.

#### 2.5.12 Determination of (Na^+^/K^+^)- and Mg^2+^- ATPase activities

Cerebrum homogenized in Tris–HCl buffer was used to determine ATPase activities (Kawamoto et al., 2005). Total ATPase activity was determined by assaying inorganic phosphate (Pi) released from hydrolyzed ATP and forming a complex with molybdate. The reaction was initiated by adding 40 μL of cerebrum homogenate to 200 μL ATPase buffer (pH 7.2) containing 3 mM ATP, 120 mM NaCl, 2 mM KCl, 3 mM MgCl_2_, and 30 mM histidine, with and without ouabain (3 mM). ATPase activity was measured after 60 min of incubation at 37°C. Reaction was achieved by the addition of a quenching solution (0.6 mL) containing 1 N H_2_SO_4_ and 0.5% ammonium molybdate. The formation of a blue phosphomolybdate complex was determined spectrophotometrically at 700 nm. A standard curve was run using H_2_PO_4._ Total ATPase activity, Mg^2+^-, and (Na^+^/K^+^)-ATPase were determined by an inorganic phosphate (Pi) assay, in which Pi is released from the enzymatic hydrolysis of ATP. Enzyme activity is expressed as μmol Pi liberated/h/mg protein.

#### 2.5.13 Plasma preparation and determination of lactate dehydrogenase activity in the cerebrum and plasma

At the end of the experimental period, the animals of different groups were sacrificed by cervical decapitation to avoid stressful conditions. Some blood samples were taken and centrifuged at 2500 rpm for 5 min to separate the plasma. Plasma samples were kept at −20°C for further LDH biochemical assays.

The determination of lactate dehydrogenase (LDH) activity in the cerebrum homogenate and plasma was assayed using a commercial reagent kit purchased from Biomaghreb (Ariana, Tunisia; Ref: 20012) and was expressed as units per gram of tissue and units per liter, respectively.

### 2.6 Molecular analysis

Total DNA was extracted according to the method described by Chomzynski (1987) and performed by the method described by Kanno et al. (2004). DNA samples (10 mg of DNA/lane) were analyzed by electrophoresis at 80 V for 1 h on a 1% agarose gel treated with ethidium bromide. The gel was visualized under an ultraviolet lamp and photographed. Band intensities were detected using Quantity One analysis software. All determinations were performed in triplicate.

### 2.7 Urine collection and corticosterone determination

Twenty-four hours before euthanasia, the animals were housed in metabolic cages to collect urine. The urine samples were used to determine corticosterone concentration (CSB-E05112r) using the enzyme-linked immunosorbent assay (ELISA) kit (Cusabio, Houston, TX, United States).

### 2.8 Histological examination

The cerebrum tissue collected from each group was randomly selected for light microscopy. Samples were fixed in formalin solution, embedded in paraffin, serially cut into 5-μm thick sections, and stained with hematoxylin–eosin (Manfred, 1968). The scoring cerebrum abnormalities were as follows: grade 0: no damage, grade 1: **<**25% damage, grade 2: 25–50% damage, and grade 3: >50% damage (Ben Saad et al., 2017).

### 2.9 Statistical analysis

Data were analyzed using the statistical package program Stat View 5 Software for Windows (SAS Institute, Berkley, CA). Statistical analysis was performed using a one-way analysis of variance followed by Fisher’s protected least significant difference test as a *post-hoc* test for comparison between groups. All values were expressed as the means ± standard deviation (SD). Differences were considered statistically significant at a value of p of ≤0.05.

## 3 Results

### 3.1 Effects of stressors on the behavior of rodent groups

#### 3.1.1 Spontaneous locomotion activity of rats

**Figure 2:**
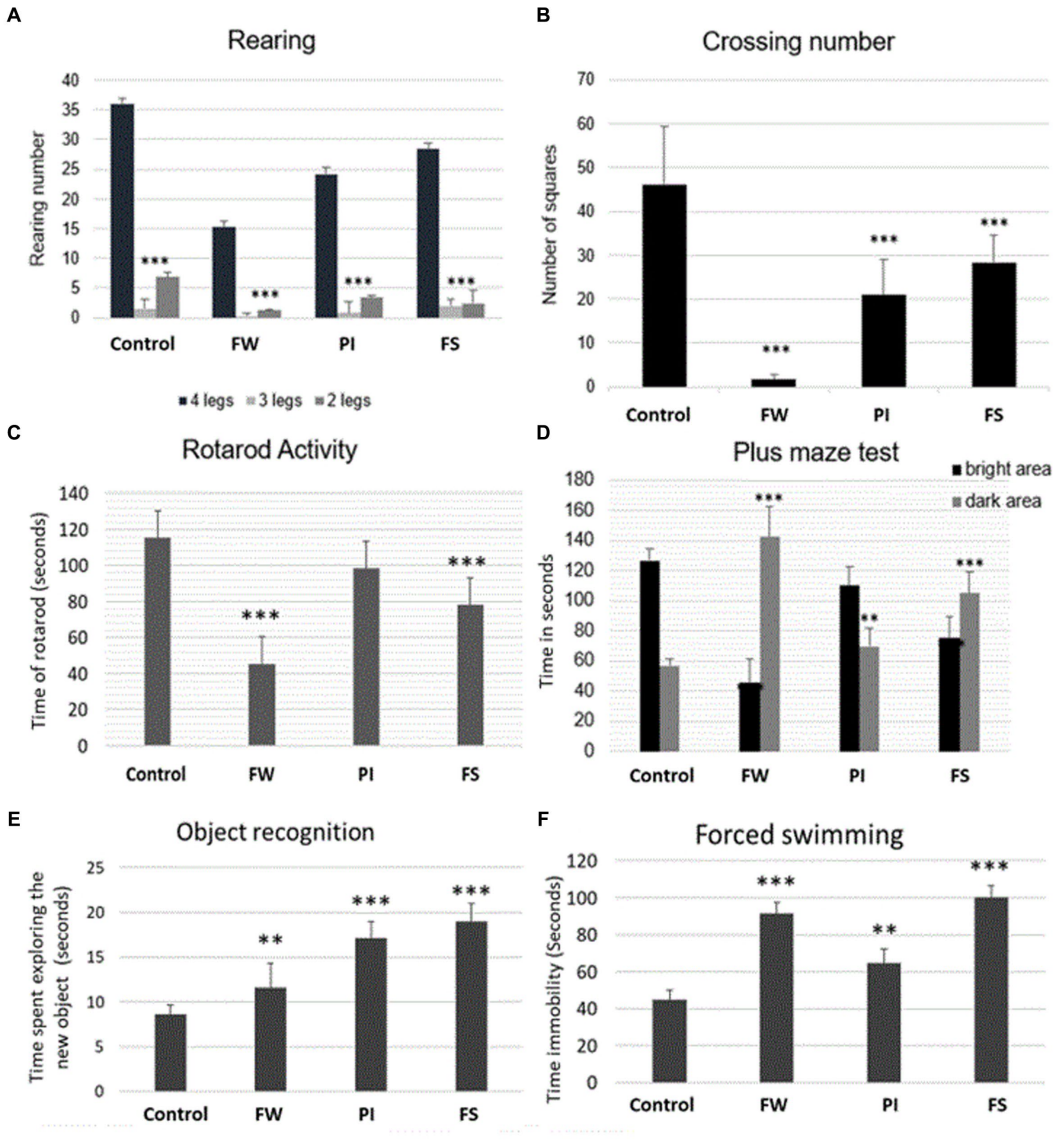
Effect of stress on rats’ muscle grip strength [rotarod test, **(A)**], mobility [open field test, **(B,C)**], anxiety [plus maze test, **(D)**], memory [object recognition test, **(E)**], and depression [forced swimming test, **(F)**]. Stressed groups vs. the control: *p < 0.05, **p < 0.01, ***p < 0.001. FW, food and water deprivation; PI, permanent illumination; FS, forced swimming.

All stressed rats showed less activity with decreased rearing (Figure 2A). The crossing number test showed a deeper decrease in mobility in the FW group (Figure 2B). Exposure to FW and FS resulted in a significant decrease in falling time compared to the control group (Figure 2C).

#### 3.1.2 Elevated plus maze test

The three stressed groups exhibited more significant latencies than the control. The maximum difference was registered in the FW group, where the time passed in the dark area was the highest compared to PI, FS, and the control group (Figure 2D).

#### 3.1.3 Working memory: object recognition

On day 1, the control group showed a lesser time for exploring the new object. On day 2, the ‘familiar’ object was spatially displaced to the corner of the open field, whereas the ‘novel’ one remained in its original location. The rats in the control group contacted the displaced object more frequently than the stationary one. The FS and PI groups contacted the displaced object significantly less frequently compared to the control group. Overall, there was a significant increase in time spent exploring new objects in all tested groups compared to the control (Figure 2E).

#### 3.1.4 Depression symptom assessment: forced swimming (Porsolt test)

After exposing rats to work stress forms (FW, PI, and FS), there was a significant increase in immobility time by the forced swim test in all tested groups compared to the control group (Figure 2F). The increase in immobility time demonstrated the depression potential.

### 3.2 Effects of stressors on the brain fatty acid composition

The fatty acid composition of the cerebrum tissue in control and stress-exposed rats is summarized in Table 1. Palmitic acid (C16:0) and stearic acid (C18:0) were the dominant saturated fatty acids in all samples. Levels of stearic acid, palmitic acid, and ω-3 were increased upon stress compared to control group linoleic acid (C18:0). Other fatty acids, including those of docosahexaenoic acid (C22:6 ω-3), palmitoleic acid (C16:1 ω-9), linolenic acid (C18:3 ω-3), arachidonic acid (C20:4 ω-6), and w-6 fatty acid, decreased in stressed rats compared to control. Polyunsaturated fatty acids (PUFAs) decreased in all groups compared to the control, with the deepest values registered in the FS group (Table 1).

**Table 1:** Changes in the fatty acid composition (%) in the cerebrum of controls and stressed rats.

Fatty acid (%)	Control	FW group	PI group	FS group
Saturated				
Palmitic acid (C 16:0)	30.00 ± 0.10	32.75 ± 0.16*	32.50 ± 0.10*	33.00 ± 0.10**
Stearic acid (C 18:0)	20.50 ± 0.05	21.75 ± 0.05*	22.50 ± 0.10*	23.00 ± 0.10**
Behenic acid (C 22:0)	03.50 ± 0.10	02.75 ± 0.01	03.50 ± 0.10*	03.50 ± 0.15*
Unsaturated				
Palmitoleic acid (C 16:1 ω-9)	00.75 ± 0.01	00.5 ± 0.01**	0.25 ± 0.01****	0.25 ± 0.01***
Oleic acid (C 18:1 ω-9)	26.00 ± 0.06	25.50 ± 0.01	25.00 ± 0.01	24.50 ± 0.01*
Linolenic acid (C 18:2 ω-6)	01.25 ± 0.01	01.00 ± 0.06**	0.75 ± 0.01***	0.75 ± 0.01***
Arachidonic acid (C 20:4 ω-6)	09.25 ± 0.01	6.25 ± 0.02***	05.50 ± 0.34***	5.00 ± 0.05***
Docosahexaenoic acid (C 22:6 ω-3)	08.00 ± 0.08	08.75 ± 0.03	08.75 ± 0.01*	08.75 ± 0.01*
Linoleic acid	00.75 ± 0.01	00.85 ± 0.01	01.25 ± 0.01***	01.25 ± 0.01***
Σ of saturated	54.00 ± 2.00	57.25 ± 0.87**	58.50 ± 2.08**	59.50 ± 1.54***
MUFA	29.00 ± 0.63	27.00 ± 1.00*	26.00 ± 1.54*	25.50 ± 2.16**
PUFA	17.00 ± 1.09	15.75 ± 0.37*	15.50 ± 0.10*	15.00 ± 0.85**
ω-3	08.50 ± 0.10	09.50 ± 0.10*	10.00 ± 0.10**	10.50 ± 0.01**
ω-6	10.50 ± 0.10	07.50 ± 0.50**	7.00 ± 1.00**	6.50 ± 0.50***
TOTAL (Saturated, MUFA, PUFA)	100	100	100	100

PUFA polyunsaturated fatty acid(s), MUFA monounsaturated fatty acid(s).

Values are expressed as mean ± standard deviation values of three replicates.

Comparison between stressed groups and control: *p < 0.05, **p < 0.01, ***p < 0.001.

### 3.3 Effects of stressors on the biochemical parameters

#### 3.3.1 Urinary corticosterone concentration

**Figure 3:**
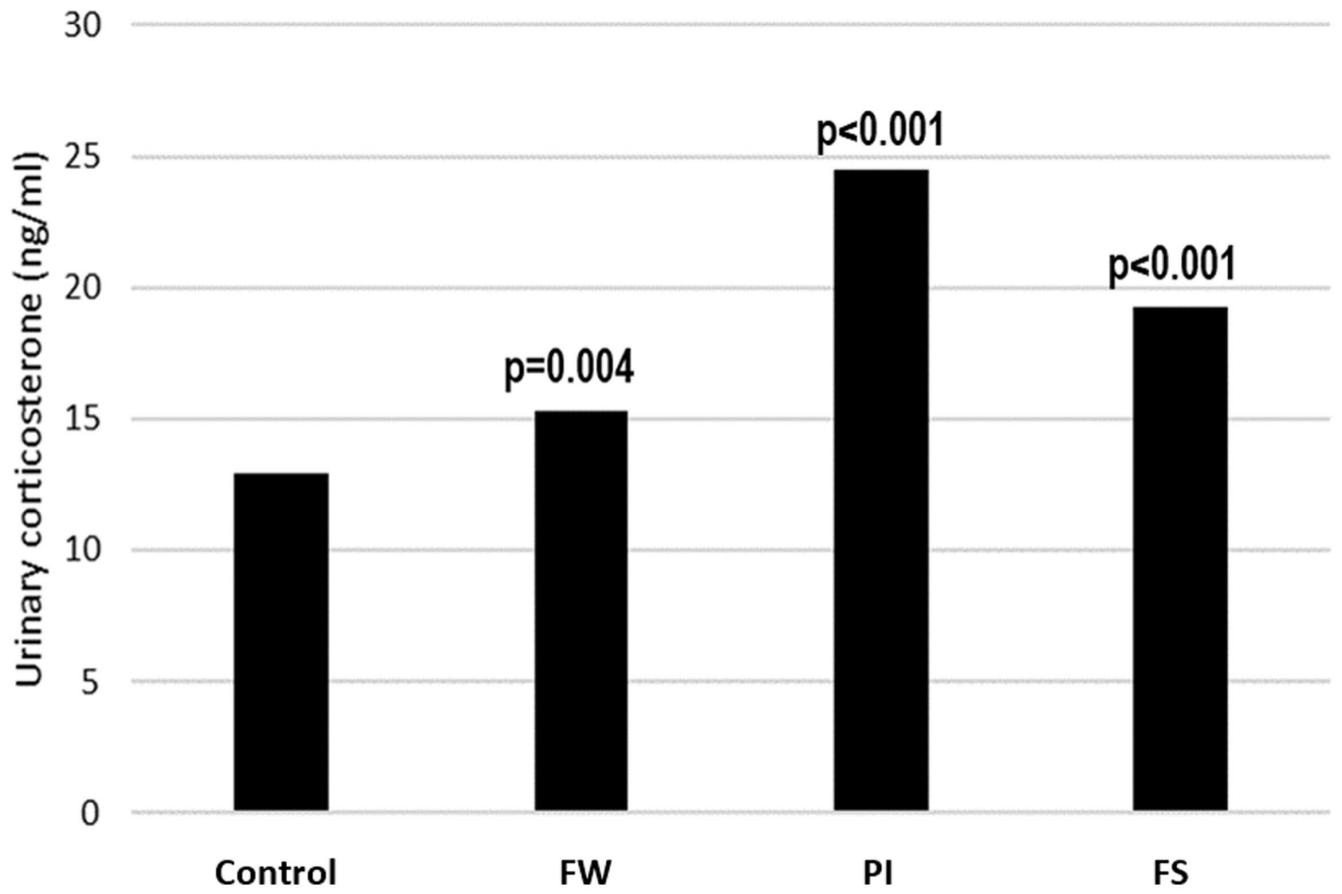
Effect of stress on urinary corticosterone. *p*-values indicate the comparison versus the control group. FW, food and water deprivation; PI, permanent illumination; FS, forced swimming.

The urinary corticosterone concentration was 12.94 ± 0.09 ng/mL in the control group and was increased in all stressed groups, with the highest level registered in the PI group. The concentration was 15.27 ± 0.30 ng/mL in the FW group, 24.51 ± 0.87 in the PI group, and 19.23 ± 0.40 in the FS group (Figure 3).

#### 3.3.2 Lactate dehydrogenase in plasma and cerebrum tissue

**Figure 4:**
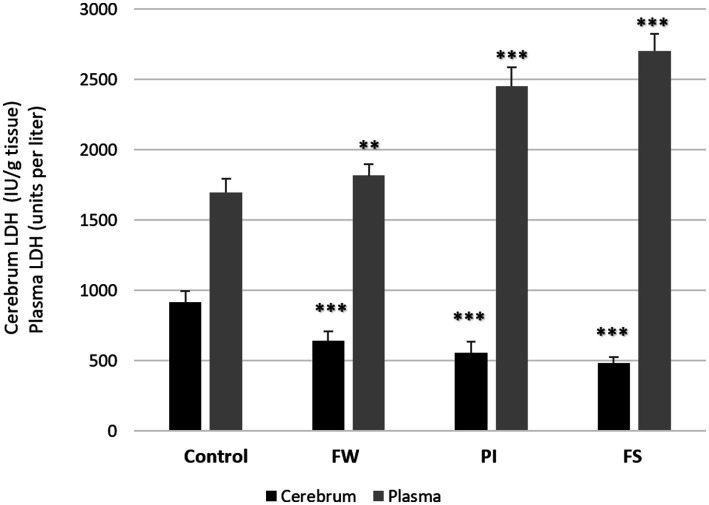
Effect of work stress on LDH activities in plasma and cerebrum tissue. Stressed groups vs. the control: *p < 0.05, **p < 0.01, ***p < 0.001. FW, food and water deprivation; PI, permanent illumination; FS, forced swimming.

LDH levels in plasma increased in all stressed groups: 2704 ± 124 IU/L for the FW group (*p* < 0.01), 2,454 ± 134 IU/L for the PI group (*p* < 0.001), and 1816 ± 79 IU/L for the FS group (*p* < 0.001) compared to the control (1,697 ± 99 IU/L). Contrarily, at the cerebrum level, LDH dosage was decreased in all exposed groups compared to the control (*p* < 0.001) (Figure 4).

### 3.4 Oxidative and antioxidative marker levels in brain tissue

Table 2 shows the impact of stress exposure on stress oxidative markers and on enzymatic and non-enzymatic antioxidant levels in the brain tissue.

**Table 2:** Impact of food and water deprivation, permanent light exposure, and forced swimming stress on stress oxidative markers and on enzymatic and non-enzymatic antioxidant levels in the brain tissue.

Parameters/Groups	Control	FW group	PI group	FS group
MDA (nmol/mg protein)	3.23 ± 0.25	6.08 ± 0.99***	8.25 ± 0.41***	9.40 ± 0.53***
LOOHs (nmol/mg protein)	6.01 ± 0.47	8.54 ± 0.73**	8.87 ± 0.95**	11.72 ± 0.75***
H_2_O_2_ (μmol/mg protein)	0.40 ± 0.04	1.02 ± 0.02***	1.29 ± 0.05***	1.51 ± 0.12***
AOPP (μmol/mg protein)	0.81 ± 0.06	1.21 ± 0.16**	1.56 ± 0.14***	1.78 ± 0.09***
PCO (nmol/mg protein)	0.02 ± 0.003	0.03 ± 0.003*	0.04 ± 0.01***	0.04 ± 0.009***
SOD (units/mg protein)	26.83 ± 1.48	19.91 ± 1.88*	17.68 ± 1.41***	13.38 ± 1.56 ***
GPx (nmol GSH/ min/mg protein)	69.72 ± 2.46	47.16 ± 1.59***	39.66 ± 2.55***	27.81 ± 2.21***
CAT (mmol H_2_O_2_ consumed/min/mg protein)	23.68 ± 1.74	16.64 ± 1.80***	13.53 ± 1.54***	12.26 ± 1.07***
GSH (nmol/mg protein)	21.73 ± 1.83	18.38 ± 1.80**	12.59 ± 0.77***	10.01 ± 1.30***

Values are expressed as mean ± standard deviation for six animals in each group.

Comparison between stressed groups and control: *p < 0.05, **p < 0.01, ***p < 0.001.

*MDA levels* were increased in all stressed groups. They were 6.08 ± 0.99 nmol of MDA/mg protein for the FW group, 8.25 ± 0.41 nmol of MDA/mg protein for the PI group, and 9.40 ± 0.53 nmol of MDA/mg protein for the FS group, while it was 3.23 ± 0.25 nmol of MDA/mg protein for the control group (*p* < 0.001 for all groups compared to the control).

*LOOH levels* were increased in the FW group (8.54 ± 0.73 nmol of LOOH/mg protein; *p* < 0.01), the PI group (8.87 ± 0.95 nmol of LOOH/mg protein; *p* < 0.01), and the FS group (11.72 ± 0.75 nmol of LOOH/mg protein; *p* < 0.001) compared to the control group (6.01 ± 0.47 nmol of LOOH/mg protein).

*AOPP levels* were increased in all stressed groups compared to the control (0.81 ± 0.06 nmol of AOPP/mg protein). They were 1.21 ± 0.16 in the FW group (*p* < 0.01), 1.56 ± 0.14 in the PI group (*p* < 0.001), and 0.81 ± 0.06 nmol of AOPP/mg protein in the FS group (p < 0.001).

*H_2_O_2_ levels* were increased in all stressed groups compared to the control (0.40 ± 0.04 μmol/mg protein). They were 1.02 ± 0.02 in the FW group (*p* < 0.001), 1.29 ± 0.05 in the PI group (*p* < 0.001), and 1.51 ± 0.12 μmol/mg protein in the FS group (*p* < 0.001).

*PCO levels* were also increased in all stressed groups compared to the control (0.02 ± 0.003 nmol/mg protein). The highest PCO variation was registered in the PI and FS groups (0.04 ± 0.01 and 0.04 ± 0.01 nmol/mg protein, respectively; *p* < 0.001 each).

*SOD levels* were 19.91 ± 1.88 units/mg protein; *p* < 0.05 for the FW group, 17.68 ± 1.41 units /mg protein; *p* < 0.001 for the PI group, and 13.38 ± 1.56 units /mg protein; p < 0.001 for the FS group compared to the control group (26.83 ± 1.48 units /mg protein).

*GPx levels* were 47.16 ± 1.59 nmol GSH/min/mg protein; p < 0.001 for the FW group, 39.66 ± 2.55 nmol GSH/min/mg protein; p < 0.001 for the PI group, and 27.81 ± 2.21 nmol GSH/min/mg protein; p < 0.001 for the FS group compared to the control group (69.72 ± 2.46 nmol GSH/min/mg protein).

*GSH levels* reported were 18.38 ± 1.80 nmol/mg protein; *p* < 0.01 for the FW group, 12.59 ± 0.77 nmol/mg protein; *p* < 0.001 for the PI group, and 10.01 ± 1.30 nmol/mg protein; *p* < 0.001 for the FS group compared to the control (21.73 ± 1.83 nmol/mg protein).

*CAT levels* were 16.64 ± 1.80 mmol H_2_O_2_ consumed/min/mg protein; p < 0.001 for the FW group, 13.53 ± 1.54 mmol H_2_O_2_ consumed/min/mg protein; p < 0.001 for the PI group, and 12.26 ± 1.07 mmol H_2_O_2_ consumed/min/mg protein; p < 0.001 for the FS group compared to the control (23.68 ± 1.74 mmol H_2_O_2_ consumed/min/mg protein).

### 3.5 Effect of stressors on total ATPase, Mg^2+^-ATPase, and (Na^+^/K^+^)-ATPase activities

**Figure 5:**
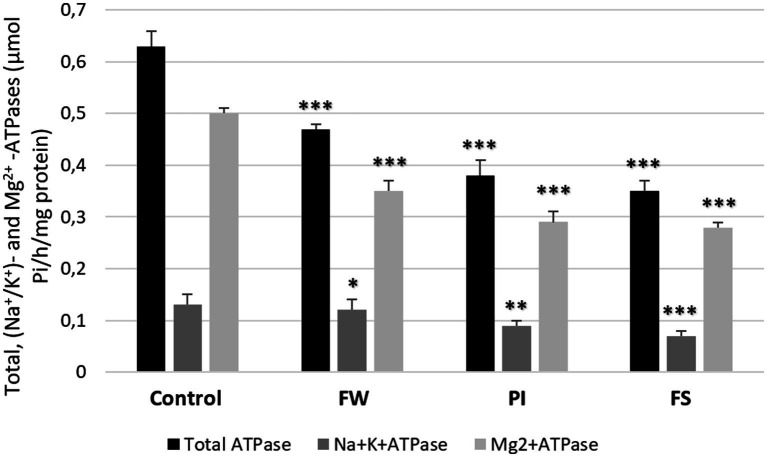
Effect of work stress on total ATPase, (Na^+^/K^+^)-ATPase, and Mg^2+^-ATPase activities in cerebrum tissue. Stressed groups vs. the control: *p < 0.05, **p < 0.01, ***p < 0.001. FW, food and water deprivation; PI, permanent illumination; FS, forced swimming.

Total ATPase, (Na^+^/K^+^)-ATPase, and Mg^2+^-ATPase activities were decreased in all stressed groups compared to the control (0.63, 0.13, and 0.5 μmol Pi/h/mg protein, respectively). In stressed rats, dosages were 0.48, 0.12, and 0.35 μmol Pi/h/mg protein for the FW group; 0.38, 0.09, and 0.29 μmol Pi/h/mg protein for the PI group; and 0.35, 0.07, and 0.27 μmol Pi/h/mg protein for the FS group. The FS group showed the most significant decrease in total ATPase, Mg^2+^-ATPase, and (Na^+^/K^+^)-ATPase activities compared to the other groups (Figure 5).

### 3.6 Effect of stressors on acetylcholinesterase activity

**Figure 6:**
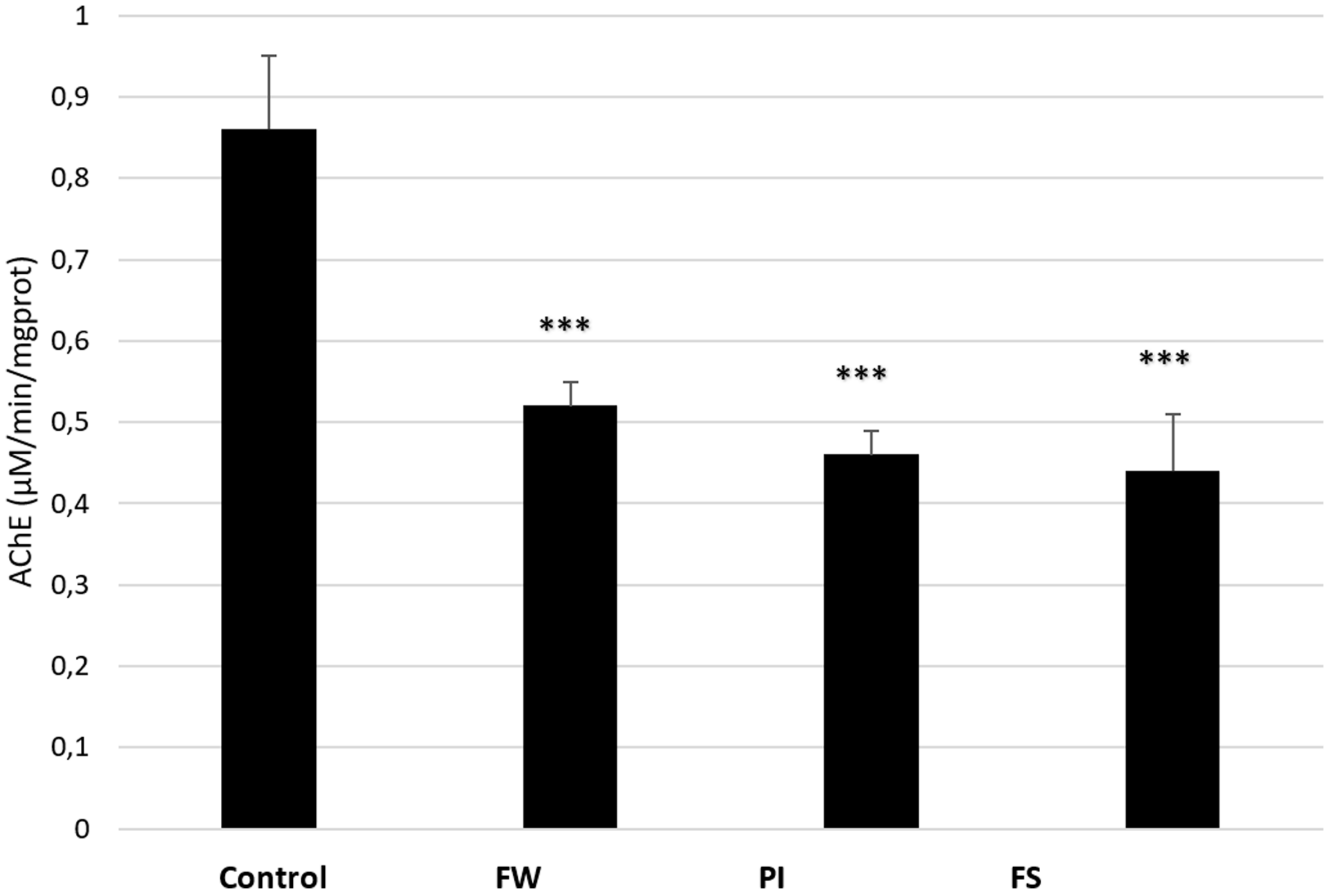
Effect of stress (food, water stress, permanent light, and forced swimming) on AChE activity in cerebrum tissue. Stressed groups vs. the control: ***p < 0.001. FW, food and water deprivation; PI, permanent illumination; FS, forced swimming.

The AChE activity in the cerebral tissue of control and stressed rats is presented in Figure 6. It was significantly decreased in all exposed groups compared to the control (*p* < 0.001 for the three groups). AChE in the cerebral tissue was 0.52 in the FW group, 0.47 in the PI group, and 0.44 μM/min/mg protein in the FS group, while it was 0.86 μM/min/mg protein in the control group.

### 3.7 Effect of stressors on DNA fragmentation

**Figure 7:**
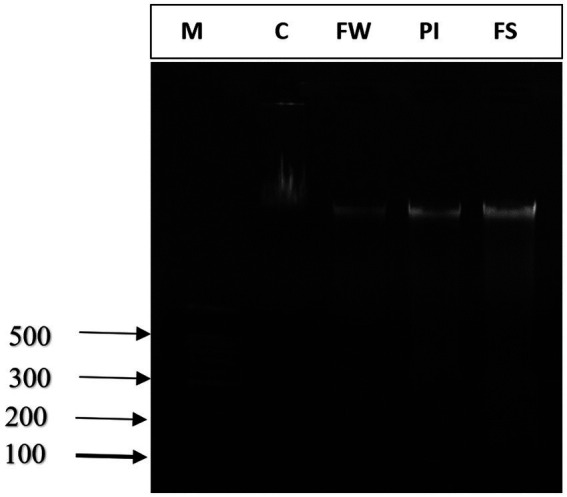
Agarose gel electrophoresis of DNA fragmentation. M: marker; lane 1: control group (C), lane 2: food and water stress group (FW), lane 3: Permanent illumination (PI) group, lane 4: forced-swimming group (FS).

Agarose gel electrophoresis showed undetectable DNA laddering in the cerebrum tissue of control rats. The intact DNA band appeared condensed near the application point with no DNA smearing, suggesting no DNA fragmentation. A smear without ladder formation on agarose gel was observed in the cerebral cells of stress-exposed rats, indicating random DNA degradation (Figure 7).

### 3.8 Histopathological changes

**Figure 8:**
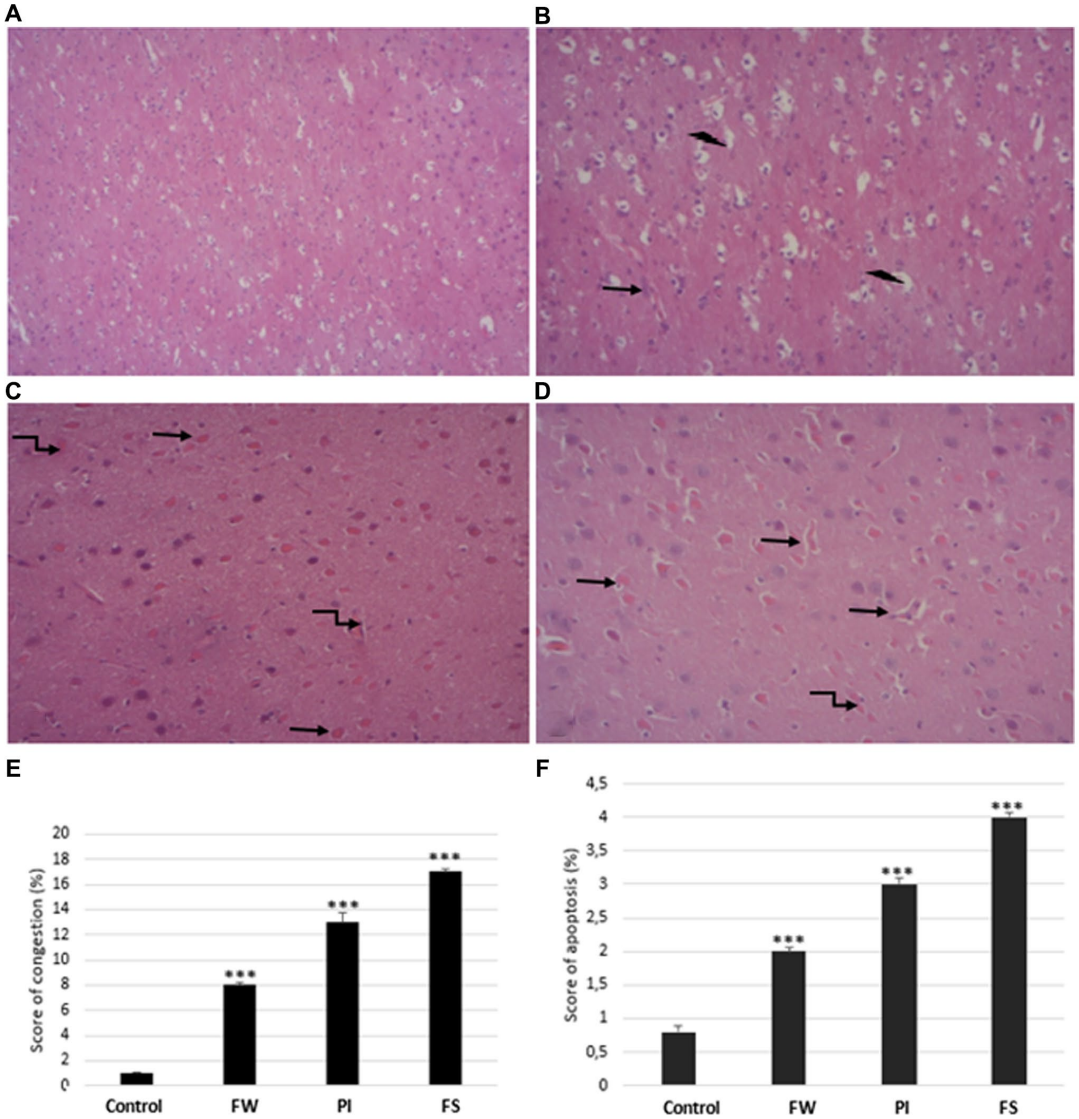
Cerebrum histological sections of controls **(A)**, food and water stress **(B)**, permanent illumination **(C)**, and forced-swimming stress **(D)** groups. **(E,F)** Histological NAS scores of cerebrum tissue: values are given as means ± SD (mean of three determinations). Stressed groups vs. the control: ***p < 0.001. Arrows indicate: 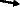 apoptosis cells, 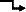 necrosis, 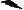 vascular congestion. Sections were stained with hematoxylin–eosin and observed with light microscopy, (x200).

The histological architecture was normal in the control rats’ cerebrum (Figures 8A–D). Exposure to stressors induces degenerative changes in the cerebrum tissue, as seen by severe distortions in cellular architecture. In the cerebrum tissue, stress exposure induced vascular congestion and apoptosis in the affected area (Figures 8E,F). These severe cerebral damages are significantly greater in the FS group. The results in the congestion and apoptosis percentage were 13 and 3% for the FW group, 8 and 2% for the PI group, and 17 and 4% for the FS group compared to the control (1 and 0.8%).

## 4 Discussion

Stress is widely described as leading to physical and mental health disorders (Ganster and Schaubroeck, 1991). It triggers biological dysregulations involving various mechanisms such as the enzymatic process (acetylcholine/anticholinesterase), the inflammatory pathway mainly cytokines, the hypothalamic–pituitary–adrenal axis (ACTH and cortisol), and a runaway of the oxidative system (ROS and RNS) (Vreeburg et al., 2009; Dowlati et al., 2010). The present study estimates the impact of working conditions on the brains of health caregivers using an animal model. It evaluates changes in behavioral, enzymatic, and hormonal activities, oxidative stress impact, and cellular damage.

The cerebrum is the brain area involved in motor control and is, therefore, intensely affected by stress conditions. Locomotor activity may influence functional outcomes in animal models of brain injury or disease. Therefore, it is important for the neuroscientist to carefully monitor the locomotor changes (decreased movements, balance, and muscle strength) associated with coordination difficulty (Curzon et al., 2009).

Locomotor tests in open-field and rotarod activities are parameters experimentally used to assess stressor-induced behavioral disorders. In our study, rats acutely exposed to stress conditions present motor impairment, as evidenced by decreased crossing and rearing movements and rotarod activities.

Anxiety is a psychological and behavioral state induced in animals and humans by stress exposure. It is characterized by fear and annoyance (Naqvi et al., 2012). The elevated plus maze is a widely used behavioral test to assess anxiety symptoms and their related behaviors (Ekeanyanwu and Njoku, 2015). The model is based on the natural dislike of rodents for open and lit spaces. In our study, anxiety symptoms were more pronounced in stressed groups with a higher time passed in the dark area compared to the control group.

Working memory does not derive from a discrete system, as do vision and motor control. Rather, working memory is a property of the brain that supports the successful attainment of behavioral goals. The latter are carried out by several systems, including sensory and motor systems, and those supporting semantic and episodic memory. The object recognition test was used as a neural mechanism that likely underlies the working memory function (Hannesson et al., 2008). In our study, the rat exposed to stressors contacted the displaced object significantly less frequently compared to the controls, confirming the decrease in memory capacity.

The forced swim test is the most commonly employed behavioral test to assess despair, which is similar to human depression (Wei-Jun, 2011). A significant number of studies have reported that rats exposed to stress exhibit depressive-like behaviors, as evidenced by increased immobility period in behavioral tests (Kumar et al., 2011). Therefore, the present data agreed with previous findings that showed consistent depressive-like behavior induced by repeated and unpredictable stress in rats.

Anxiety- and depression-like behaviors are well correlated with AChE inhibition (McCloskey et al., 2017). Indeed, the inhibition of AChE in the central and peripheral nervous systems results in ACh accumulation and excessive activation of muscarinic and nicotinic receptors (McDonough and Shih, 1997). ACh is involved in providing adaptative responses to metabolic and environmental changes and to peripheral body alerts (Kaizer et al., 2004; Belujon and Grace, 2011). High levels of ACh are implicated in the pathophysiology of depression (Suarez-Lopez et al., 2019; Abdulla and Picciotto, 2023). A recent study showed a link between exposure to chronic stress and neuronal dysregulation in the lateral nucleus of the tegmentum, particularly in those producing ACh (Fernandez et al., 2018). In addition, the selective blocking of these neurons’ activity during stress exposure can prevent the appearance of behavioral disorders (Fernandez et al., 2018). In the present study, acetylcholinesterase (AChE) activity decreased in the brain and plasma of stressed rats. In this context, the pathophysiological mechanism of AChE regulation is multifactorial. Some authors highlighted cross-links between ROS production and AChE activity (Deb and Das, 2021). Accordingly, some ROS, such as H_2_O_2_ and peroxides, could inhibit AChE activity, while OH hampers AChE activity in the rat brain. In the present study, we documented significant apoptosis and DNA damage levels in all exposed groups. Moreover, brain fatty acids play a major role in ACh biosynthesis regulation. Thus, in the presence of significant cellular damage, a decrease in the biosynthesis of ACh in the rat’s brain could lead to a downward modulation of AChE since its activity level directly depends on the presence of ACh at post-synaptic receptors (Ben Saad et al., 2017).

Cortisol is a primary stress hormone for neurohumoral responses and behavioral changes in humans, while it is corticosterone in many animals such as amphibians, reptiles, rodents, and birds (Nandam et al., 2019). Fonken and Nelson reported that exposure to light stress could increase cortisol levels and affect the circadian system (Fonken and Nelson, 2014). In humans, ACTH and cortisol are produced in response to different stressors (Fonken and Nelson, 2014). Peaks occur between 20 and 40 min after onset (Dickerson et al., 2004), with some interindividual differences. Elevated cortisol levels are generally correlated with decreased activity in the prefrontal cortex (Harrewijn et al., 2020; Dziurkowska and Wesolowski, 2021). At the same time, prolonged amygdala activation leads to an increased inability to manage emotion and is responsible for negative thoughts (De Raedt and Koster, 2010). These level variations may be linked to the background of each individual. Thus, past stress experiences, personality, psychological state during stressful events, or education may influence everyone’s ability to anticipate or adapt (Pulopulos et al., 2018). These elements can affect the amplitude of the neurobiological response and, consequently, the risk of diseases related to increased cortisol levels. Finally, hypercortisolemia can be associated with affective disorders leading to major depression. In our study, the urine levels of corticosterone in stressed groups were significantly higher than in the control group. In addition, the submission to sustainable stress (21 days) allowed the tested groups to develop different coping strategies depending on the exposure time and the intensity of the stressor. Clearly, the FS group was under the most intense stress. It had to provide the most demanding physical and emotional strain while having the least time to adapt to the stressful situation, which caused the highest corticosterone release. This result confirms that anticipatory cognitive appraisal influences the magnitude of the stress-induced cortisol response (Salzmann et al., 2018). Moreover, in acute stress, the dynamic response leads first to a high level of ACTH release. Then, ACTH falls to baseline levels, but cortisol secretion continues to pulsate, indicating increased sensitivity of the adrenal cortex to ACTH (Russell and Lightman, 2019). Thus, knowledge of the threat seems to be anticipated with a hormonal response involving a faster release of cortisol during a new exposure, which suggests an adaptive physiological phenomenon.

Oxidative stress is involved in many diseases, including neurological and mental disorders (Bhatt et al., 2020). Redox turbulences generate lipid peroxidation (LPO), protein, and DNA alteration in the brain. ROS and RNS mainly target lipids in several steps involving prooxidant and antioxidant agents. When oxidative damage exceeds the restorative abilities of the human body, lipid peroxidation alters the structure of the cellular membrane and is responsible for the loss of its biofunctions. Lipid peroxidation results from free radical-mediated oxidation, non-radical and non-enzymatic lipid peroxidation, and enzymatic oxidation (Niki, 2009). Thus, levels of hydroperoxides, nitro-fatty acids, oxysterols, and aldehydes may vary in many diseases. Stress exposure enhances oxidative stress in the cerebrum with significant histological changes (McDonald and Windebank, 2002; Wang et al., 2021). Moreover, cerebrum cell apoptosis correlates well with emotional strain in animals and humans (Bachis et al., 2008; Atrooz et al., 2021). In the present study, severe distortions in the cellular architecture were observed under microscopic examination. Stress exposure provoked neuronal degeneration and encephalomalacia. Congestion and apoptosis were more marked in the FS group.

Brain fatty acids play a significant role in ACh biosynthesis regulation, contributing to normal cognitive function (Moreno et al., 2004). Polyunsaturated fatty acids (PUFAs) are abundant in the nervous system and represent a privileged target for free radicals due to their molecular structure. They perform vital functions such as boosting synaptogenesis and neurogenesis, inducing antinociception, and stimulating gene expression and neuronal activity (Deplanque, 2004; Pathan et al., 2008), thus improving cognitive performance (Rinwa et al., 2010). PUFAs, including ω-3, such as linolenic acid, and ω-6, such as arachidonic acid, are essential for brain development and functioning. In the present study, all stressed groups presented fatty acid composition changes, especially decreased brain arachidonic acid and ω-6 levels. The ω-6 PUFAs regulate cellular functions, including differentiation, proliferation, cell cycle signaling, and apoptosis (Zarkovic, 2003). The PUFA changes in the cerebral cells’ biomembrane can destroy the special arrangement and impair local enzyme activities, including ATPases. Given that ATPase activities may undergo a series of changes under stress conditions, they are considered a sensitive toxicity indicator. This study recorded a significant decrease in the (Na^+^/K^+^)-ATPase and Mg^2+^-ATPase activities after stress exposure. Hence, decreasing enzymatic activities can lead to the selective suppression of sustained neuronal firings, excitatory synaptic transmission, and neuronal dysfunction (Ben Saad et al., 2017). Therefore, the changes in the brain fatty acid levels might be induced by oxidative stress, as demonstrated by an increase in free radical generation and MDA, AOPP, LDH, and LOOH levels, mainly in the FS group. The main primary lipid peroxidation product is LOOH, while MDA is the most mutagenic product. It is a biomarker of lipoperoxidation resulting from arachidonic and larger PUFA degradation and is generated by enzymatic and non-enzymatic processes (Ayala et al., 2014). MDA constitutes a reliable biomarker of oxidative stress *in vivo* (Giera et al., 2012). Physical exercise can increase oxidative stress *in vivo* (Alessio et al., 2000; Ciocoiu et al., 2007). An increased number of carbonyl residues accompany oxidative damage to proteins after work-related stress exposure. A significant increase in protein carbonyl levels in the FS group was noted compared with the other stressed groups. Finally, we found LDH disturbance, known to amplify ROS in certain circumstances linked to cellular hydrogen peroxide production (Wu et al., 2021).

In our experiment, stress exposure resulted in massive DNA fragmentation and subsequent DNA smear formation on agarose gel. The enhanced DNA oxidation was closely related to increased ROS and lipid peroxidation products. The antioxidant defense systems of the living body consist of antioxidant enzymes that may be involved in reducing oxidative stress (Ben Saad et al., 2017). Work is known to have differential effects on antioxidant enzymes (Salminen et al., 1984), depending on the mode and intensity of exercise (Wu et al., 2008). In the present study, all prooxidant markers increased significantly compared to the control group. The highest variation level was registered in the FS group. Additionally, the decrease in the antioxidant enzyme level in the cerebral tissue was more pronounced in the FS than in the PI and FW groups.

Our study has several limitations. First, this is an experimental study on animals with extrapolation to work conditions in humans. Second, we used three different stressors in three different groups of animals without experimenting with the effect of cumulative exposure. Third, we have probably not measured some parameters that can affect neurological and redox changes. However, our model allowed us to investigate stressful conditions such as those encountered in hospital wards (permanent light, water and food restriction, physical strain, and peaks of stress exposition). Some of these strains’ impact has already been investigated in the clinical setting, but our study is the first experimental report to make the link between stressful conditions and stress at work in hospitals. It also links the intensity and duration of the stressor with its consequences for health. Finally, this study can open avenues to investigate the effects of work-related strains and oxidative stress on behavior and neurological changes at the caregiver’s level.

## 5 Conclusion

The present study on an animal model examines the working conditions of caregivers in health facilities. It shows that the combination of different stress factors to which caregivers are exposed daily has an undeniable neuropsychological impact. We also highlighted that the oxidative response is correlated with the duration and intensity of the exposure. Thus, short and intense stress revealed higher oxidative biomarkers than long and lower-intensity exposure. By analogy, work stress can be responsible for neurological and psychological disorders. It can impact the performance of caregivers and, therefore, the quality of care. This experimental series pleads for extending research to the human level. It also calls for awareness among hospital managers to put in place strategies for prevention and wellbeing at work.

## Data availability statement

The raw data supporting the conclusions of this article will be made available by the authors, without undue reservation.

## Ethics statement

The animal study was approved by Ethics Committee of the “Higher Institute of Biotechnology, University of Sfax – Tunisia” (Protocol no. 09.0010/22). The study was conducted in accordance with the local legislation and institutional requirements.

## Author contributions

JP: Conceptualization, Formal analysis, Methodology, Writing – original draft, Writing – review & editing. DF: Validation, Writing – review & editing. HB: Investigation, Resources, Writing – review & editing. MG: Investigation, Resources, Writing – review & editing. AD: Investigation, Resources, Writing – review & editing. MaM: Investigation, Resources, Writing – review & editing. AB: Validation, Writing – review & editing. RM: Validation, Writing – review & editing. SH: Validation, Writing – review & editing. AR: Validation, Writing – review & editing. MuM: Validation, Writing – review & editing. FN: Validation, Writing – review & editing. BdT: Validation, Writing – review & editing. IB: Conceptualization, Formal analysis, Methodology, Writing – original draft, Writing – review & editing. HK: Conceptualization, Formal analysis, Methodology, Writing – original draft, Writing – review & editing.
